# Novel Antibiotic Combinations of Diverse Subclasses for Effective Suppression of Extensively Drug-Resistant Methicillin-Resistant *Staphylococcus aureus* (MRSA)

**DOI:** 10.1155/2020/8831322

**Published:** 2020-10-29

**Authors:** Shumyila Nasir, Muhammad Sufyan Vohra, Danish Gul, Umm E Swaiba, Maira Aleem, Khalid Mehmood, Saadia Andleeb

**Affiliations:** ^1^Department of Industrial Biotechnology, Atta-ur-Rahman School of Applied Biosciences (ASAB), National University of Sciences and Technology (NUST), Islamabad 44000, Pakistan; ^2^Department of Pharmacy, Abbottabad University of Science and Technology, Havelian, Pakistan

## Abstract

The emergence of multidrug-resistant pathogens such as methicillin-resistant *Staphylococcus aureus* (MRSA), the chief etiological agent for a range of refractory infections, has rendered all *β*-lactams ineffective against it. The treatment process is further complicated with the development of resistance to glycopeptides, primary antibiotics for treatment of MRSA. Antibiotic combination therapy with existing antimicrobial agents may provide an immediate treatment option. Minimum inhibitory concentrations (MICs) of 18 different commercially available antibiotics were determined along with their 90 possible pairwise combinations and 64 triple combinations to filter out 5 best combinations. Time-Kill kinetics of these combinations were then analyzed to find collateral bactericidal combinations which were then tested on other randomly selected MRSA isolates. Among the top 5 combinations including levofloxacin-ceftazidime; amoxicillin/clavulanic acid-tobramycin; amoxicillin/clavulanic acid-cephradine; amoxicillin/clavulanic acid-ofloxacin; and piperacillin/tazobactam-tobramycin, three combinations were found to be collaterally effective. Levofloxacin-ceftazidime acted synergistically in 80% of the tested clinical MRSA isolates. First-line *β*-lactams of lower generations can be used effectively against MRSA infection when used in combination. Antibiotics other than glycopeptides may still work in combination.

## 1. Introduction

Hospital-acquired methicillin-resistant *Staphylococcus aureus* (MRSA) has been a predominant agent for skin and nosocomial infections for several years [1–3]. MRSA is responsible for about more than 20% of all bloodstream infections, and mortality rate is as high as 25–50% [4, 5]. Methicillin resistance in *Staphylococcus aureus* is mediated by SCC*mec* gene, which encodes polypeptide penicillin-binding-protein 2a (PBP2a) [6, 7] and also provides insertion sites for plasmids and transposons which assist in transmission of resistance to non-*β*-lactam antibiotics [8, 9]. Emergence of *β*-lactamase-producing MRSA strains has rendered all *β*-lactams ineffective against its infections. Glycopeptides (mainly vancomycin) remain the major class of antibiotics for treatment of MRSA, but indiscriminate use of these antimicrobial agents has led to the emergence of vancomycin-resistant *S. aureus* (VRSA) [10]. Development of resistance and other complications such as infection recurrence, and treatment failure pose a serious hindrance in treatment of MRSA infections [4, 11]. Therefore, potential options for treatment of these refractory infections need to be explored. Since other therapeutic interventions such as phage therapies are still in development, antibiotic combination therapy with existing antimicrobial agents provides an immediate treatment option.

Usage of multidrug combinations has proven successful against infection caused by *H. pylori*, *M. tuberculosis*, *A. baumannii*, *P. aeruginosa*, and *K. pneumonia* [12–16]. Historically, vancomycin-aminoglycoside combination was successfully employed for endocarditis caused by MRSA, but due to increased possibility of renal impairment, this combination is not recommended anymore [17–19]. More recent studies, where vancomycin was used concurrently with beta-lactams improved in vitro results, were observed. In another study, antibiotics from different subclasses and generations of *β*-lactams (meropenem, piperacillin, and tazobactam) were successfully combined against MRSA infections [20].

In this study, we combined FDA approved antibiotics from different generations and classes to determine synergistic combinations which do not necessarily include vancomycin (or any other glycopeptide) against highly resistant MRSA. We anticipate that using antibiotic combinations of commercially available antibiotics from diverse subclasses has a potential for overcoming antibiotic-resistant infections and may serve as a powerful technique in reversing antibiotic resistance on top of producing bactericidal effects.

## 2. Materials and Methods

**Figure 1:**
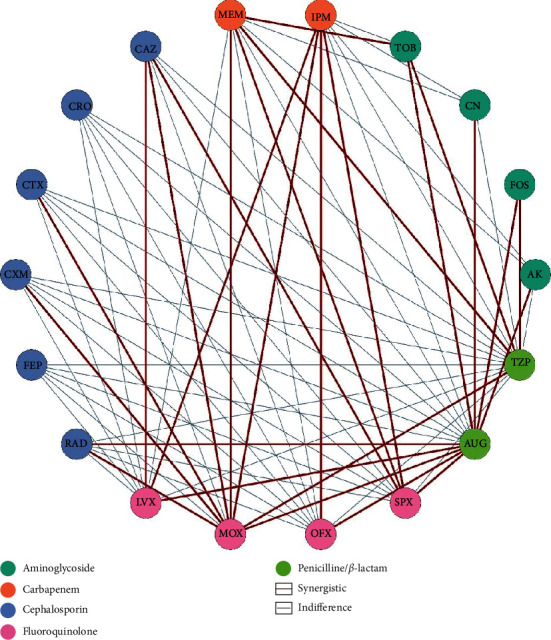
Pairwise antibiotic testing against MRSA. (a) List of pairwise combinations. (b) MIC by broth microdilution indicates that synergistic relationship exists between moxifloxacin and lower-generation cephalosporin (FICI 0.14–0.265). (c) Levofloxacin-ceftazidime shows FICI value as low as 0.281 indicating synergy. (d) Meropenem demonstrates synergy with higher generation of fluoroquinolones, i.e., moxifloxacin (FICI 0.132) and sparfloxacin (FICI 0.265). (e) Imipenem shows collateral sensitivity with all fluoroquinolones (FICI 0.126 each).

**Figure 2:**
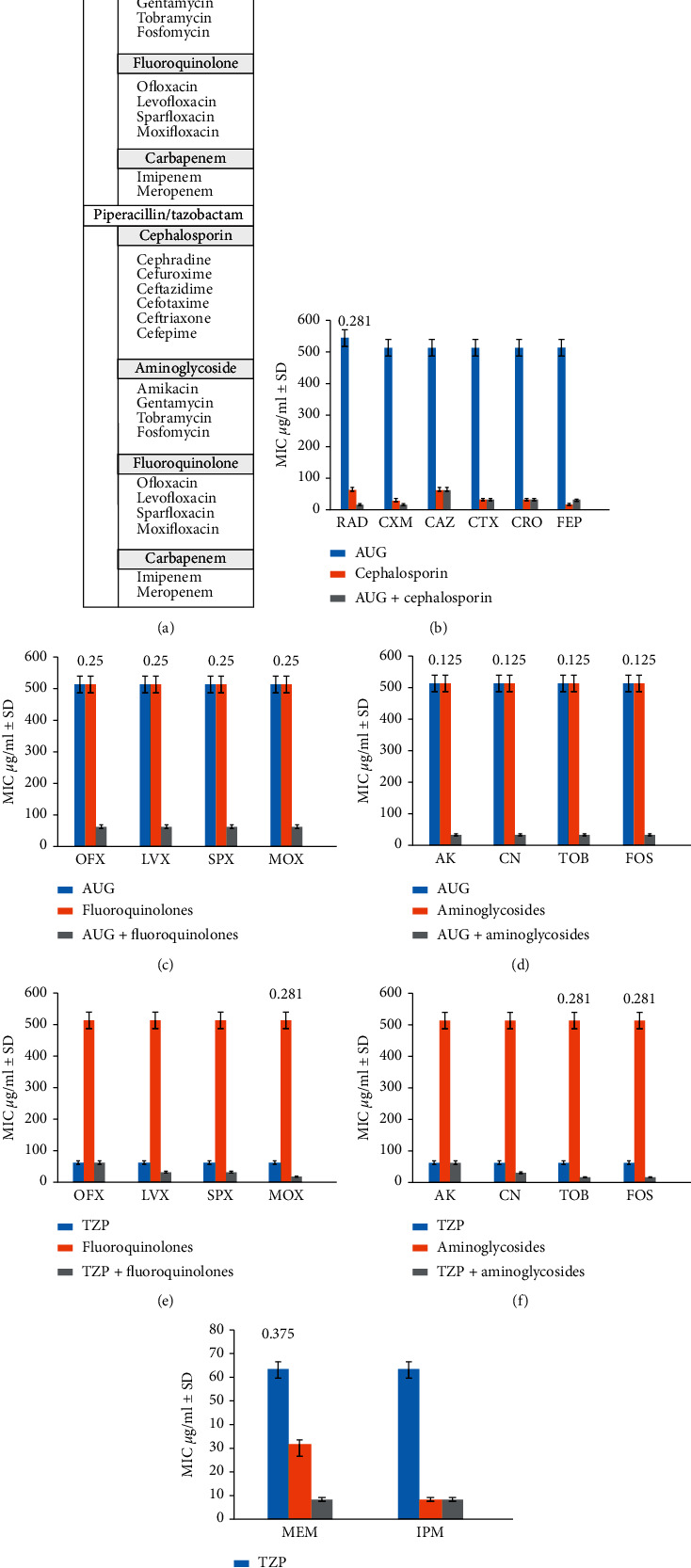
Triple antibiotic testing against MRSA. (a) List of triple combinations. (b) Amoxicillin-clavulanic acid-cephalosporin MIC indicates that synergistic relationship exists only between amoxicillin-clavulanic acid-cephradine (FICI 0.281). (c) MIC of amoxicillin-clavulanic acid-fluoroquinolone combination shows that synergy exists in all combinations (FICI 0.25 each). (d) A significant decrease in MIC of amoxicillin-clavulanic acid-aminoglycoside in combinations (FICI 0.125). (e) Reduction in combined MIC is only observed with moxifloxacin in piperacillin-tazobactam-fluoroquinolone combinations (FICI 0.281). (f) Significant activity is observed when piperacillin-tazobactam is combined with higher-generation aminoglycoside, i.e., tobramycin (FICI 0.281) and fosfomycin (FICI 0.281). (g) Meropenem in combination with piperacillin-tazobactam exhibits synergy (FICI 0.375).

Isolates of *S. aureus* were acquired from major hospitals of Rawalpindi, Lahore, and Peshawar, and then screened for methicillin and vancomycin resistance. Further susceptibility testing by the Kirby disk diffusion method was performed, and by using clinical breakpoints from the Clinical and Laboratory Standards Institute (CLSI), an extensively drug-resistant (XDR) MRSA isolate (LR-2) was selected for antibiotic synergy testing [21]. A total of 18 different commercially available antibiotics (Table 1) were used for 90 possible pairwise combinations (Figure 1(a)) and 64 triple combinations (antibiotic from varying class/penicillin/inhibitor) (Figure 2(a)). Each experiment was conducted in duplicate, and mean values were calculated for each combination.

XDR MRSA isolate was cultured in cation-adjusted Muller-Hinton Broth (MHB) for 24 hours at 37°C. Minimum inhibitory concentration (MIC) was determined by the broth microdilution method as described by Wiegand et al. [22]. Stock solutions of antimicrobial agents were prepared by calculating the amount of antibiotic to be used: (amount of drug in each tablet (y))/10 mg = (amount of active agent in each tablet)/(amount of drug needed to produce 10 mg/ml solution (x)).

The antibiotic-containing media were serially diluted in 96-well plate to create an array of 10-fold dilution across the row. Log-phase culture of MRSA was diluted in MHB to produce 1/50 dilution of bacterial suspension and inoculated in the 96-well plate. Column 11 and 12 were kept as negative and positive control, respectively. Plates were incubated for 24 hours at 37°C. Wells showing no visible turbidity after 24 h were subcultured on plate count agar for colony counting. Minimum inhibitory concentration (MIC) was considered as the lowest concentration inhibiting 99% of bacterial growth. Each experiment was performed in duplicate. Synergistic combinations were determined by the fractional inhibitory concentration index (FICI) method [23–26]. FICI calculation involves MIC of the antimicrobial agents in combination divided by the MIC of the agents alone to determine antibiotic interactions, as shown in the following formula:

(1) $$\begin{array}{c} \text{FICI}=\;\frac{{\text{MIC}}_{\left(\text{comb }AB\right)}}{\text{MIC}{\;}_{\left(A\right)}}+\;\frac{{\text{MIC}}_{\left(\text{comb6 }B\right)}}{{\text{MIC}}_{\left(B\right)}}, \end{array}$$

whereby FICI ≤0.5 was interpreted as “synergistic,” FICI = 1–4.0 was considered “indifferent,” and FICI >4.0 was inferred “antagonistic.”

**Figure 3:**
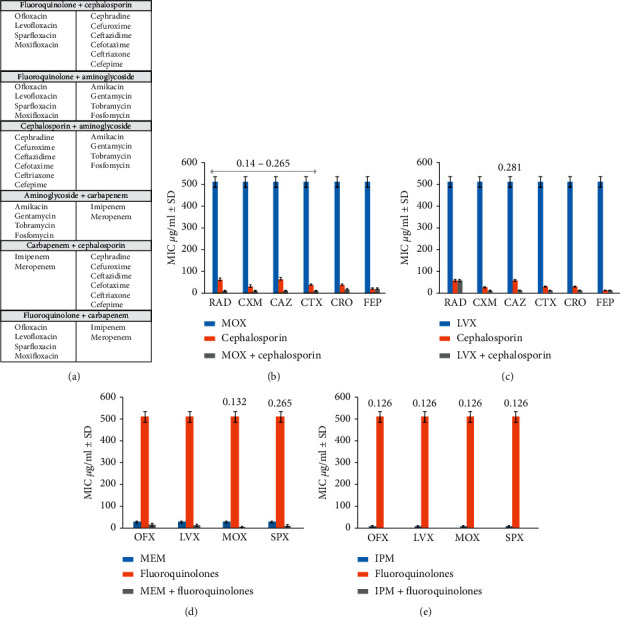
Time-Kill kinetics on antibiotics individually and collaterally. (a) Levofloxacin-ceftazidime combination with 8-fold growth reduction compared to most active monotherapy. (b) <2-log 10 difference in killing with amoxicillin-clavulanic acid-cephradine combination therapy compared to most active single treatment shows lack of synergy. (c) Amoxicillin-clavulanic acid-ofloxacin combination is collaterally indifferent as decrease in bacterial growth is <2-log 10. (d) Amoxicillin-clavulanic acid-tobramycin combination shows synergy because of >2-log 10 growth reduction. (e) Collateral sensitivity is shown in piperacillin-tazobactam-tobramycin combination.

For further analyses, five highly synergistic combinations against MRSA isolate (LR-2) were selected. The criteria for selection required antibiotics in combination to belong to lower generation with simple mechanism of action, and low FICI values. To further confirm the synergistic mechanism of action, Time-Kill assays were performed on selected combinations according to a formerly described protocol [27] (Figure 3). Briefly, MHB was inoculated with test organism and incubated to midlog phase. Aliquots containing antibiotic combination (two or three drug combination) and test MRSA at density of 10^6^ CFUs were incubated at 37°C. At times 0 h, 2 h, 4 h, 6 h, and 8 h, the inoculum was withdrawn, serially diluted from 10^3^ to 10^7^, and 100 *μ*l of each dilution was plated on a nutrient agar medium and incubated at optimal growth condition of *S. aureus* to determine colony counts [27]. Killing kinetics were interpreted as “synergistic” when ≥2-log10 reduction in growth was observed in the colony forming units (CFU) at 24 h with antibiotic combination compared to most active drug alone, and “indifferent” when <2-log10 decrease was observed.

Synergistic drug combinations were then tested on other randomly selected methicillin-resistant *S. aureus* isolates, and their MIC and FICI values were determined to validate drug combination synergy.

All assays were conducted in duplicate, and mean MIC values were calculated along with standard deviations. The FICI values were calculated from the mean MIC values obtained in individual and combination assays. The standard deviation is represented in graphs on the error bars.

## 3. Results

**Figure 4:**
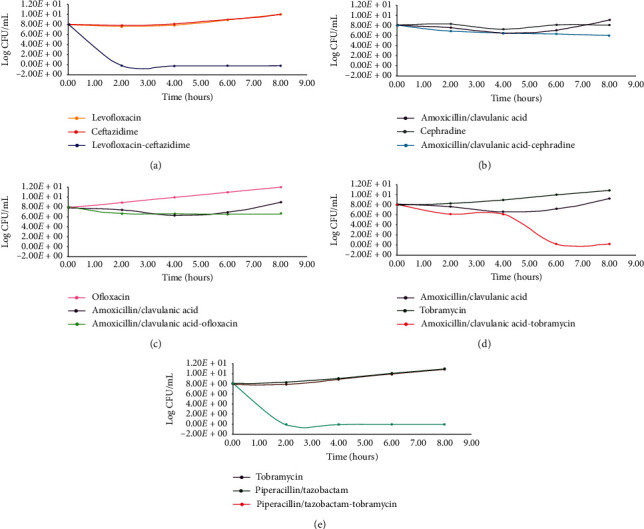
Collateral relationship network of antibiotic combinations from diverse subclasses on MRSA LR-2.

Results obtained from the Kirby–Bauer disk diffusion method on MRSA isolate (LR-2) showed complete resistance to penicillin, cephalosporin, fluoroquinolones, aminoglycosides, and glycopeptides and partial resistance to carbapenem, tetracycline, and certain miscellaneous agents. Therefore, MRSA isolate (LR-2) was classified as XDR [28]. Individual MIC assays showed insensitivity to all antibiotics from Table 1 except for imipenem (MIC = 4 *μ*g/ml). Double and triple antibiotic combinations from diverse subclasses were tested against MRSA LR-2 to identify the combinations that were synergistic when administered concomitantly.  Figure 4 shows the interactions between antibiotics indicating synergistic or indifferent relationship.

All pairwise combinations of cephalosporin with aminoglycosides or carbapenem did not exhibit a synergistic interaction. However, when combined with fluoroquinolones, some synergy was observed especially with moxifloxacin (MOX/RAD FICI = 0.140; MOX/CXM FICI = 0.265; MOX/CAZ FICI = 0.140; MOX/CTX FICI = 0.265) (Figure 1(b)). It must be noted that moxifloxacin is a higher generation antibiotic than levofloxacin [29]. An interesting pairwise synergistic combination of cephalosporin and fluoroquinolone with low FICI (0.281) was observed between CAZ/LVX (both lower generation antibiotics) (Figure 1(c)). All double antibiotic combinations of carbapenem were unsuccessful except for when combined with fluoroquinolones. Meropenem showed synergy with higher generation of fluoroquinolones (sparfloxacin and moxifloxacin with FICI of 0.132 and 0.256, respectively) (Figure 1(d)), and fluoroquinolones-imipenem combination showed a completely synergistic interaction (FICI = 0.126) (Figure 1(e)). All pairwise combinations with aminoglycosides were indifferent.

Triple combination of amoxicillin/clavulanic acid with cephalosporin yielded a peculiar result when synergy was observed with cephradine (1^st^ generation cephalosporin) with an FICI value of 0.281 (Figure 2(b)). This finding was interesting because if proven successful in further experimentation, an ineffective lower generation antibiotic [30] can be brought in use again. Sensitivity was observed when amoxicillin/clavulanic acid was combined with fluoroquinolones (FICI 0.25) (Figure 2(c)) and aminoglycosides (FICI = 0.125) (Figure 2(d)). Triple combinations of piperacillin/tazobactam with moxifloxacin showed synergy (FICI = 0.281) (Figure 2(e)), and with aminoglycosides, namely, tobramycin (FICI = 0.281) and fosfomycin (FICI = 0.281) (Figure 2(f)), piperacillin/tazobactam, and meropenem combination also showed synergy (FICI = 0.375).

The combinations selected for further testing included levofloxacin-ceftazidime; amoxicillin/clavulanic acid-tobramycin; amoxicillin/clavulanic acid-cephradine; amoxicillin/clavulanic acid-ofloxacin; and piperacillin/tazobactam-tobramycin. Levofloxacin combination with cephalosporin was selected instead of moxifloxacin combination because it is a lower-generation gluoroquinolone. Amoxicillin/clavulanic acid and cephradine was selected because both antibiotics other than the inhibitors are of lower generation. Amoxicillin/clavulanic acid showed FICI values lower than 0.5 with fluoroquinolones and aminoglycosides, so one combination from each group was selected. Ofloxacin in combination to amoxicillin/clavulanic acid made this list because it is the lowest-generation aminoglycoside. However, tobramycin though is not a low generation aminoglycoside but showed good potential with other penicillin-*β*-lactam inhibitor combinations. Hence, tobramycin in amalgamation with amoxicillin/clavulanic acid and with piperacillin-tazobactam was selected.

Time-Kill kinetics illustrate up to 8-fold reduction in growth compared to most active drug alone which means a synergistic relation exists between levofloxacin-ceftazidime; amoxicillin/clavulanic acid-tobramycin; and piperacillin-tazobactam-tobramycin (Figures 3(a)–3(e)). Bactericidal effect was observed within 8 hours of incubation in all three combinations.

The combination showing >2-log10 reduction when tested on other randomly selected MRSA isolates, levofloxacin-ceftazidime combination, was the most successful combination because collateral sensitivity was observed in 80% of MRSA isolates. Both amoxicillin/clavulanic acid-tobramycin; piperacillin-tazobactam-tobramycin combination showed synergy in 50% MRSA isolates.

## 4. Discussion

Rapid emergence of extensive drug resistance in Gram-positive and Gram-negative bacteria necessitates identification of novel therapeutic approaches. Reliance on monoantibiotic therapeutic approach provided selection pressure for development of resistance. With the depleting arsenal of antibiotics and a gap in the successful development of alternative therapeutics, determining synergistic combination of currently available antibiotics could provide an effective alternative to curtailing the alarming increase in antibiotic resistance [31].

The purpose of this study was to find combinations from diverse subclasses of antibiotics that can produce synergistic effects in combinations against extremely drug-resistant MRSA isolates. We report that a novel combination of levofloxacin-ceftazidime (LVX/CAZ) acts synergistically to produce bactericidal effect on XDR MRSA isolate LR-2. Previously, fluoroquinolone-cephalosporin combination was shown to have synergistic effect against *P. aeruginosa* [32].

Our findings on pairwise combinations suggest that fluoroquinolones (especially levofloxacin and moxifloxacin) are very effective when combined with cephalosporin and carbapenem antibiotics. Though monotherapy with fluoroquinolones is recommended to be minimized for treatment of *S. aureus* infections because of multidrug resistance [33], this finding provides a solution to overcome fluoroquinolone resistance in MRSA.

The overall higher sensitivity ratios to amoxicillin/clavulanic acid in combination with other antibiotics is not surprising since existing evidence on antimicrobial combinations suggests that despite being the first-line antibiotic, beta-lactam antibiotics in combination therapy are crucial for the treatment of obstinate *S. aureus* bacteremia [34]. Besides, amoxicillin/clavulanic acid is recommended in amalgamations to overcome vancomycin or daptomycin resistance in MRSA [35–37]. Amoxicillin/clavulanic acid in conjunction with cephradine, an ineffective lower-generation antibiotic against MRSA [30], was an interesting combination. This lower-generation antibiotic proved successful in Time-Kill studies and shows the potential to be used again as a therapeutic agent in combination. Although, amoxicillin/clavulanic acid-tobramycin trio showed >2-log10 decrease in XDR MRSA isolate (LR-2), it did not show synergistic interactions in other isolates. Amoxicillin/clavulanic acid/fluoroquinolone (ciprofloxacin) combination has also been proven successful before for treatment of Gram-negative infections of prosthetic joints [38]. However, it must be noted that most of these isolates showed low individual MIC values for tobramycin and amoxicillin/clavulanic acid, and so the FICI values were mostly “indifferent.” Synergy between piperacillin-tazobactam-meropenem combination is consistent with the findings of PR Gonzales and his colleagues [20].

The inability to confirm all synergistic combinations by Time-Kill assay was a limitation of our study. Due to this, we might have overlooked certain combinations that might produce better results than the ones we screened. However, the main objective of our study to test and identify combinations from diverse subclasses that interacted synergistically, belonged to lower generations, produced bactericidal effects, and overcome the resistance for first-line antibiotics that could not be used anymore was achieved.

Although coadministration of drugs is a promising approach to mitigate rapid evolution of antibiotic resistance, it may also lead to production of unexpected and unwanted outcomes as reported previously [39]. Although the FIC index was determined to ensure that a synergistic relationship exists between selected antibiotics when used in combination, further investigations may help optimize the management of antibiotic combinations.

## 5. Conclusion

We report that pairwise LVX/CAZ combination allows us to use first-line antibiotics that not only produce bactericidal effects against XDR MRSA but also address need of time by extending the lifespan of existing antibiotics. This combination will be immediately available for use since these drugs have already been approved by the FDA. Resistance to antibiotic combination though inevitable, evidence-based synergistic studies might diminish the emergence of resistance. For future prospects, we propose that these combinations should be further tested in vivo to determine if these amalgams are as effective in vivo as they were in vitro. We also suggest that investigations should be carried out on how these combinations, where target proteins of the antibiotics involved in amalgam, work synergistically at molecular and systemic level to give a better insight. This will help predict better and robust combinations in future.

## Figures and Tables

**Table 1:** List of antibiotics tested against the MRSA isolate (LR-2).

Antibiotic	Brand name	Manufacturer	Form	Potency	Final conc.
Cephalosprin					
Cephradine (RAD)	Velosef	GlaxoSmithKline (GSK)	IV	1 g	10 mg/ml
Cefuroxime (CXM)	Zinacef	GlaxoSmithKline (GSK)	IV	1.5 g	10 mg/ml
Ceftazidime (CAZ)	Fortum	GlaxoSmithKline (GSK)	IV	500 mg	10 mg/ml
Cefotaxime (CTX)	Claforan	Sanofi-Aventis Pak Ltd.	IV	1 g	10 mg/ml
Ceftriaxone (CRO)	Rocephin	F.Hoffmann-La Roche Ltd.	Tablets	1 g	10 mg/ml
Cefepime (FEP)	Maxipime	GlaxoSmithKline (GSK)	IV	1 g	10 mg/ml

Aminoglycosides					
Amikacin (AK)	Gracil	Sami Pharmaceticals (Pvt.) Ltd.	IV	500 mg/2 ml	10 mg/ml
Gentamycin (CN)	Genticyn	Ray Pharma (Pvt.) Ltd.	IV	80 mg/2 ml	10 mg/ml
Tobramycin (TOB)	Nebcin	AGP Limited	IV	80 mg/2 ml	10 mg/ml
Fosfomycin (FOS)	Monural	Zambon	Sachet	3 g	10 mg/ml

Fluoroquinolones					
Ofloxacin (OFX)	Oflobid	Hilton Pharma (Pvt.) Ltd.	Tablets	200 mg	10 mg/ml
Levofloxacin (LVX)	Leflox	Getz Pharma	Tablets	500 mg	10 mg/ml
Sparfloxacin (SPX)	Sparaxin	Abbot Laboratories (Pak) Ltd.	Tablets	100 mg	10 mg/ml
Moxifloxacin (MOX)	Avelox	Bayer Pharma AG			10 mg/ml

Carbapenem					
Meropenem (MEM)	Meronem	Pfizer Limited	IV	500nmg	10 mg/ml
Imipenem (IPM)	Tienam	Merck Sharp & Dohme Corp. (MSD)	IV	500 mg	10 mg/ml

Ampicillin + *β*-lactam Inhibitor					
Amoxicillin/clavulanic acid (AUG)	Augmentin	GlaxoSmithKline (GSK)	Tablets	625 mg	10 mg/ml
Piperacillin/tazobactam (TZP)	Tanzo	Bosch Pharmaceuticals (Pvt.) Ltd	IV	4.5 g	10 mg/ml

## Data Availability

Data produced and analyzed during this study are included in this article.

## Abbreviations

MRSA: Methicillin-resistant *Staphylococcus aureus*
XDR: Extremely drug resistant
VRE: Vancomycin-resistant *Enterococcus* species
FDA: Food and Drug Administration
MIC: Minimum inhibitory concentration
FICI: Fractional inhibitory concentration index
MHB: Muller-Hinton Broth
CLSI: Clinical and Laboratory Standards Institute
RAD: Cephradine
CXM: Cefuroxime
CAZ: Ceftazidime
CTX: Cefotaxime
CRO: Ceftriaxone
FEP: Cefepime
AK: Amikacin
CN: Gentamycin
TOB: Tobramycin
FOS: Fosfomycin
OFX: Ofloxacin
LVX: Levofloxacin
SPX: Sparfloxacin
MOX: Moxifloxacin
MEM: Meropenem
IPM: Imipenem
AUG: Amoxicillin/clavulanic acid
TZP: Piperacillin/tazobactam.
